# Evidence for a causal relationship between psoriasis and cutaneous melanoma: a bidirectional two-sample Mendelian randomized study

**DOI:** 10.3389/fimmu.2023.1201167

**Published:** 2023-07-12

**Authors:** Nana Zhao, Pengsen Guo, Mei Tang, Fan Yang, Tongtong Zhang, Rui Mao

**Affiliations:** ^1^ Department of Operating Room, The Third People’s Hospital of Chengdu, Chengdu, China; ^2^ The Center of Gastrointestinal and Minimally Invasive Surgery, The Third People’s Hospital of Chengdu, Chengdu, China; ^3^ Emergency Department, Peking University Third Hospital, Peking University School of Medicine, Beijing, China; ^4^ Medical Research Center, The Third People’s Hospital of Chengdu, The Affiliated Hospital of Southwest Jiaotong University, The Second Chengdu Hospital Affiliated to Chongqing Medical University, Chengdu, Sichuan, China; ^5^ Department of Dermatology, Xiangya Hospital, Central South University, Changsha, China

**Keywords:** bidirectional two-sample mendelian randomization, psoriasis, cutaneous melanoma, genome-wide association study, SNP

## Abstract

**Background and objective:**

Existing cross-sectional and retrospective studies were unable to establish a causal relationship between psoriasis and cutaneous melanoma (CM). We sought to evaluate the causal role between psoriasis and CM.

**Methods:**

We performed a bidirectional two-sample Mendelian randomization analysis using summary statistics from genome-wide association studies of psoriasis and CM among individuals of predominantly European ancestry. Mendelian randomization–Egger regression, inverse variance weighting, Mendelian Randomization Pleiotropy RESidual Sum and Outlier, weighted mode, and weighted median were used to examine the causal effect between psoriasis and CM.

**Results:**

Genetically predicted psoriasis was a significant risk factor for CM (odds ratio, 1.69; 95% confidence interval, 1.15–2.48; P = 0.025). In contrast, no association was observed between genetically predicted CM and psoriasis.

**Conclusion:**

Our findings corroborated the existence of genetically predicted psoriasis increases risk of CM. Enhanced early screening of cutaneous melanoma in patients with psoriasis may improve clinical burden. However, we did not find evidence for a causal link from CM to psoriasis, so further studies are required to elucidate the effect of CM activity on psoriasis.

## Introduction

At present, there is controversy regarding whether the risk of CM in patients with psoriasis is higher than that in the general population (1–5). Although the chronic inflammatory state in psoriasis patients may induce carcinogenic effects, psoriasis treatments like systemic therapy, ultraviolet (UV) radiation, and tumor necrosis factor (TNF)-α therapy (6–10) may also put psoriasis patients at greater risk for CM. In addition, studies on psoriasis risk in patients with CM are few in number and inconsistent (11–13).

Traditional observational epidemiological studies face many challenges in discovering disease etiology and inferring causality, such as reverse causal associations, potential confounding factors, minor exposure factors, and multiple tests. In observational and retrospective cohort studies, it is difficult to explore a causal relationship between psoriasis and CM with the existence of treatment or other interfering factors beyond our control. Prospective randomized controlled trials or other research methods that can rule out these interfering factors are urgently needed to establish a causal relationship between psoriasis and CM.

In recent years, with the continuous development of statistical methods, large-sample genome-wide association study (GWAS) data, epigenetics, and various “omics” techniques, the Mendelian randomization (MR) study design has been increasingly widely used in the discussion of the causal association between complex exposure factors and disease outcomes (14–16). The research design of MR follows the Mendelian inheritance law of “parental alleles are randomly assigned to offspring.” If the genotype determines the phenotype, the genotype is associated with the disease through the phenotype, so the association between psoriasis and CM can be inferred using the genotype as an instrumental variable. Notably, MR is less susceptible to confounding factors because germline genetic variations are randomly allocated during meiosis and thus can capture exposure without being influenced by reverse causality. As a variant of the MR approach, bidirectional MR can be applied to ascertain the causal direction between two associated phenotypes. In this study, we performed a bidirectional two-sample MR analysis using summary statistics of large GWASs from the FinnGen Consortium to assess the causal association between psoriasis and CM.

## Materials and methods

### Data sources

The GWAS summary statistics for psoriasis were derived from data published by FinnGen Consortium R9. This study used the “psoriasis” phenotype. The GWAS of psoriasis included 373,338 Finnish adult subjects, including 9,267 cases and 364,071 controls, which excluded subjects with other cancers. Age, sex, top 10 major components, and genotyping batches were corrected during analysis. As a genetic instrumental variable, the GWAS summary data of CM came from the two-stage genome-wide meta-analysis conducted by Matthew et al. (17) that included 13 GWAS datasets from Europe, Australia, the United Kingdom, Athens, and the United States encompassing a total of 15,990 melanoma cases and 26,409 controls.

### Instrumental variable

We use the following five criteria to select instrumental variables (IV). First, the SNP–phenotype association level must reach a genome-wide significance threshold (P< 5*10^−8^). Second, the linkage disequilibrium between all SNPs is based on the European 1000 Genomes Project reference panel. Third, SNPs with a secondary allele frequency (MAF) of ≤ 0.01 were removed. Fourth, among the SNPs where R^2^< 0.001 (clumping window size = 10,000 kb), only those with the lowest P value were retained. Finally, when palindromic SNPs existed, we used the allele frequency information to infer the forward-strand alleles. We used a curated genotype-phenotype database (PhenoScanner) to search for associations between variants used to detect each instrumental variable and other traits that may represent pleiotropic pathways; specific examples are traits associated with hypertension and telomere length, which are recognized risk factors for cutaneous melanoma (18, 19). Variants associated with these and other traits were excluded from sensitivity analysis (using a software default threshold of P< 5 × 10 – 8).

### Statistical analysis

In the present study, we evaluated the causal relationship between psoriasis and CM using various methods, including inverse variance weighting (IVW), MR–Egger regression, MR Pleiotropy RESidual Sum and Outlier (MR-PRESSO), weighted mode, and weighted median. IVW, also known as the inverse variance weighting method, is characterized by ignoring the existence of an intercept term in regression and using the reciprocal of outcome variance (se^2^) as the weight for fitting. In the absence of heterogeneity and horizontal pleiotropy, IVW estimates are the most reliable (20). MR–Egger regression is conducted under the assumption of InSIDE (instrument strength is independent of the direct effect), which enables us to assess the presence of poly-efficacity with intercept terms. The intercept term being 0 implies that horizontal pleiotropy is absent, and the result of MR–Egger regression is consistent with IVW (21). In addition, MR–Egger regression is also used to judge whether there is horizontal pleiotropy or not. When more than 50% of the instrumental variables are invalid, the weighted median method can estimate causality more correctly. The weighted model estimation method can better detect the causal effect, has less deviation, and a lower class I error rate than the MR–Egger regression when the InSIDE hypothesis is not met (22). In addition, significant outliers were detected using MR-PRESSO (23) tests and MR–Egger regression, and horizontal pleiotropic effects were corrected by removing outliers. We used the global test to check if horizontal pleiotropy among all instruments existed (23). Moreover, we applied Cochran’s Q-test statistics to further examine the heterogeneity among all SNPs. We identify potentially heterogeneous SNPs by conducting a “leave-one-out” analysis by excluding each instrument SNP in turn. Finally, we also performed reverse MR analysis of psoriasis and CM. We adopted methods and settings that were consistent with the forward MR.

The F statistic was calculated by the formula $F=\left(\frac{R^{2}}{1-R^{2}}\times \frac{\left(n-k-1\right)}{k}\right)$ (where R^2^ is the proportion of the variance of the trait explained by the SNP, k is the number of IVs, and N is the sample size of the GWAS of the SNP with the trait), which is used to quantify the strength of the instrument, and a value > 10 is considered sufficient. We used the “TwoSampleMR” R package to calculate the coefficient of determination (R^2^) of exposure to genetic variants. The R^2^ value was estimated using the formula $R^{2}=2\times EAF\times \left(1-EAF\right)\times {\left(\beta \right)}^{2}$ (where EAF is the effect allele frequency [EAF] of SNP, SD is the standard deviation, and β is the estimated effect size of SNP on the trait). Based on the online MR-power calculation tool (https://sb452.shinyapps.io/power/) (24), we calculated the power of the MR estimates.

All analyses were performed in R (version 4.2.2; R Foundation for Statistical Computing, Vienna, Austria), and MR analysis was conducted based on the TwosampleMR (25) (version 0.5.6) and MR-PRESSO (version 1.0) R packages.

## Results

### Psoriasis to CM

**Figure 1:**
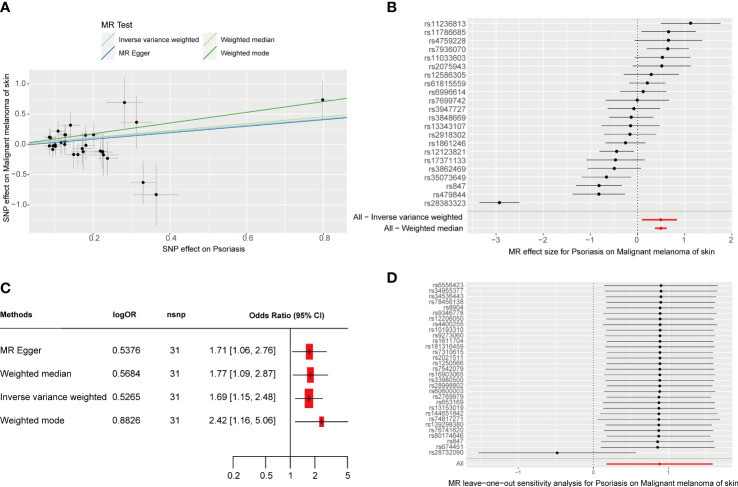
MR analysis of the causal relationship between genetically predicted psoriasis and CM. **(A)** Scatterplots for the causal association between genetically predicted psoriasis and CM. The slope of a straight line indicates the magnitude of causality. Black dots represent genetic instruments included in the main MR analysis. **(B)** Forest map visualization of the causal impact of each SNP on CM risk. **(C)** Forest mapping used four methods to visualize the causal effects of psoriasis on CM risk. **(D)** “Leave-one-out” plots for the causal association between genetically predicted psoriasis and CM.

As shown in **Table 1**, we identified a total of 31 independent SNPs as instrumental variables in psoriasis, explaining 29.22% of the total variation. The F-statistic range for SNPs was 31.9–1018.0, indicating that SNPs explain the potency strength of exposure effectively. **Figures 1A, B** show the effect of each SNP locus on CM. Furthermore, the results of IVW estimation suggest that psoriasis is an independent risk factor for CM (odds ratio [OR], 1.69; 95% confidence interval [CI], 1.15–2.48; P = 0.025; **Figures 1A, C**). In addition, the results of the other three estimate methods, including weighted mode (OR, 2.42; 95% CI, 1.16–5.06; P = 0.026), MR–Egger regression (OR, 1.71; 95% CI, 1.06–2.76; P = 0.038), and weighted median (OR, 1.77; 95% CI, 1.09–2.87; P = 0.043), further validated the results of IVW estimation.

**Table 1.** Instrumental variables used in MR analysis of the association between psoriasis and CM.

exposure	SNP	Effect allele	Other allele	MAF	F	R^2^ of exposure	Exposure (psoriasis)			Outcome (CM)		
							Beta	SE	P-value	Beta	SE	P-value
psoriasis	rs10193310	A	G	0.248	55.501	0.006	0.127	0.017	9.341E-14	0.054	0.166	0.747
psoriasis	rs12206050	T	A	0.320	41.025	0.004	0.101	0.016	1.502E-10	-0.006	0.154	0.970
psoriasis	rs1250566	A	G	0.408	33.638	0.004	-0.089	0.015	6.639E-09	-0.111	0.146	0.448
psoriasis	rs13153019	C	T	0.273	38.114	0.004	0.102	0.016	6.673E-10	-0.033	0.162	0.836
psoriasis	rs139298380	A	G	0.028	56.899	0.005	0.313	0.042	4.589E-14	0.365	0.442	0.409
psoriasis	rs144651842	A	G	0.078	40.923	0.004	0.172	0.027	1.584E-10	-0.070	0.271	0.796
psoriasis	rs1611704	T	C	0.345	217.349	0.023	0.225	0.015	3.426E-49	-0.120	0.153	0.433
psoriasis	rs16903065	A	C	0.116	34.184	0.004	-0.141	0.024	5.014E-09	-0.317	0.226	0.161
psoriasis	rs181316459	C	G	0.047	102.654	0.010	0.330	0.033	3.992E-24	-0.629	0.354	0.076
psoriasis	rs2021511	T	C	0.265	40.027	0.005	-0.109	0.017	2.505E-10	-0.219	0.162	0.176
psoriasis	rs2769979	C	T	0.605	39.928	0.004	-0.096	0.015	2.635E-10	0.025	0.146	0.862
psoriasis	rs28732090	G	C	0.058	1018.007	0.070	0.798	0.025	1.000E-200	0.735	0.308	0.017
psoriasis	rs28998802	A	G	0.184	73.995	0.008	0.161	0.019	7.834E-18	-0.168	0.185	0.362
psoriasis	rs33980500	T	C	0.072	78.437	0.008	0.238	0.027	8.260E-19	-0.232	0.278	0.404
psoriasis	rs34536443	C	G	0.030	35.369	0.005	-0.282	0.047	2.728E-09	-0.692	0.418	0.098
psoriasis	rs34955377	A	G	0.116	83.401	0.008	0.202	0.022	6.697E-20	0.159	0.223	0.475
psoriasis	rs4400255	A	T	0.099	58.398	0.006	0.181	0.024	2.140E-14	-0.016	0.241	0.947
psoriasis	rs60600003	G	T	0.102	39.937	0.004	0.150	0.024	2.623E-10	-0.167	0.237	0.480
psoriasis	rs653169	G	A	0.570	32.578	0.004	-0.086	0.015	1.145E-08	0.025	0.146	0.862
psoriasis	rs6556423	T	C	0.642	139.728	0.015	-0.180	0.015	3.054E-32	-0.152	0.150	0.310
psoriasis	rs674451	C	T	0.344	68.473	0.007	0.128	0.015	1.286E-16	0.158	0.150	0.294
psoriasis	rs7310615	G	C	0.586	32.924	0.004	-0.087	0.015	9.584E-09	-0.119	0.145	0.414
psoriasis	rs74817271	A	G	0.074	70.719	0.007	0.225	0.027	4.119E-17	-0.128	0.272	0.636
psoriasis	rs7542079	C	T	0.562	39.763	0.004	0.095	0.015	2.867E-10	-0.084	0.144	0.563
psoriasis	rs76741620	G	A	0.058	32.732	0.003	0.174	0.030	1.058E-08	-0.120	0.308	0.696
psoriasis	rs78456138	T	C	0.020	38.134	0.005	-0.365	0.059	6.606E-10	0.830	0.513	0.106
psoriasis	rs80174646	T	G	0.046	35.310	0.005	-0.227	0.038	2.812E-09	0.174	0.350	0.619
psoriasis	rs847	C	T	0.635	64.721	0.007	0.127	0.016	8.628E-16	0.158	0.150	0.292
psoriasis	rs8904	A	G	0.388	66.919	0.008	-0.126	0.015	2.829E-16	0.002	0.147	0.992
psoriasis	rs9273060	T	A	0.249	174.059	0.018	0.219	0.017	9.618E-40	-0.110	0.168	0.512
psoriasis	rs9346778	T	C	0.164	31.988	0.004	-0.117	0.021	1.551E-08	-0.030	0.192	0.875

Cochran’s IVW and MR–Egger Q test showed no significant heterogeneity among these IVs (**Table S1**). Moreover, no significant change in the estimated causal effect was observed when we excluded each SNP individually during the “leave-one-out” analysis (**Figure 1D**). Therefore, the estimated effect cannot be explained by any single SNP. In addition, according to the MR–Egger regression intercept analysis (**Supplemental Table 2**) and MR-PRESSO global test (**Supplemental Table 3**), there was no significant horizontal pleiotropy. After setting the type I error rate as 0.05 and the outcome variable as binary, we obtained an MR-power value of 100%. The above results prove that the results of our MR analysis are reliable and have high efficiency.

### CM to psoriasis

**Figure 2:**
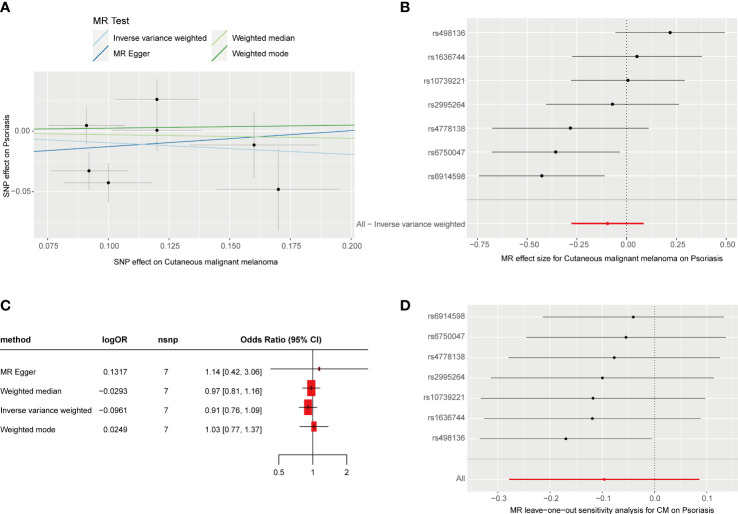
MR analysis of the causal relationship between genetically predicted CM and psoriasis. **(A)** Scatterplots for the causal association between genetically predicted CM and psoriasis. The slope of a straight line indicates the magnitude of causality. Black dots represent genetic instruments included in the main MR analysis. **(B)** Forest map visualization of the causal impact of each SNP on psoriasis risk. **(C)** Forest mapping uses four methods to visualize the causal effects of CM on psoriasis risk. **(D)** “Leave-one-out” plots for the causal association between genetically predicted CM and psoriasis.

We obtained seven SNPs (P< 5 * 10^-8^, R^2^< 0.001) significantly associated with CM from a meta-analysis of large GWASs, which explain 3% of the total variation. Summaries and details of each SNP are provided in **Supplementary Table 4**. The F-statistic range for SNPs was 30.1–49.5. The individual SNP effects and the combined effects of each method are shown in **Figures 2A, B**. The results of the four MR estimate methods all suggested that no association was observed between genetically determined CM and psoriasis (**Figure 2C**, P > 0.05). No notable heterogeneity was detected by Cochran’s Q statistics (**Supplementary Table 5**, P > 0.05). The MR–Egger intercept test (**Supplementary Table 6**) and MR-PRESSO global test (**Supplementary Table 7**) suggested no evidence of pleiotropy. Moreover, no significant change in the estimated causal effect was observed when we excluded each SNP individually during the “leave-one-out” analysis (**Figure 2D**).

## Discussion

To our knowledge, this study was the first attempt to explore the causal relationship between psoriasis and CM using the summary statistics of FinnGen Alliance R9 and the large GWAS meta-analysis conducted by Matthew et al. (17). The results of our bidirectional two-sample MR study suggested that a genetic predisposition to psoriasis was associated with a 69% increased risk of CM (hazard ratio, 1.69), while a genetic predisposition to CM was not. In addition, we used a variety of methods to validate the reliability and efficacy of our results.

Most current studies on the effect of psoriasis on CM are observational cross-sectional studies or retrospective cohort studies, with inconsistent findings. For instance, Egeberg et al.’s (1) retrospective cohort study based on a Danish population showed a significantly increased risk of CM in patients with mild psoriasis. Reddy et al.’s (3) retrospective cohort study also confirmed this finding (hazard ratio, 1.53). Nonetheless, a systematic review and meta-analysis of 365 studies found no significant association between psoriasis and CM risk (5). Important confounding factors like phototherapy and systemic anti-psoriasis therapy always influence the overall conclusions of the study. However, our study—independent of confounding factors and reverse causality—showed that genetic liability to psoriasis is a risk factor for CM. The chronic inflammatory state of psoriasis may impair immune surveillance and lead to possible tumor development, resulting in a higher risk of CM in psoriasis patients. Many of the transcription factors and cytokines—which include interleukin (IL)-6, TNF-α, and signal transduction and transcription activator 3 (26),—that are thought to play a role in psoriasis may also contribute to tumor development. Signal transduction and transcriptional activator 3 signaling is significantly elevated in keratinocytes in psoriatic lesions (27), and TNF-α has been shown to be critical for skin canceration (28). An intracellular transmitter of inflammatory signals, nuclear factor kappa-light-chain-enhancer of activated B-cells (NF-kB), is activated by pro-inflammatory cytokines such as TNF and IL-1 (29).

NF-κB has pleiotropic properties in the surrounding environment, but three are its basic roles. First, it is involved in pro-inflammatory responses. In human melanoma, many NF-κB-regulated chemokines are expressed at high levels: CXCL8 or IL8, CXCL1 (melanoma growth-stimulating activity), CCL5 (regulatory activation, normal T expression and secretion), and CCL2 (30–32). These NF-κB-regulated chemokines are thought to enhance melanoma progression through autocrine and paracrine loops upon transcriptional activation. In fact, overexpression of CXCL8 leads to metastatic tumor growth in normal primary melanoma cells and is associated with the transition from RGP to VGP in melanoma (33, 34). Second, NF-κB is also a major anti-apoptotic factor that induces the transcription of a variety of anti-apoptotic proteins, such as Bcl-XL, tumor necrosis factor receptor-related factors 1 and 2 (TRAF1 and TRAF2, respectively), and inhibitor-apoptotic (IAP) proteins 1 and 2 (c-IAP1 and c-IAP2, respectively). In advanced melanoma, upregulated NF-κB also enhances above anti-apoptosis molecules (35, 36). Finally, NF-κB promotes cell growth: activated NF-κB enhances the expression of cyclin D1, an important regulator of cell cycle progression. NF-κB upregulated Myc and cycle regulator proteins, cyclin D1, and cyclin-dependent kinase 2, which further promoted melanoma growth (37–40). Activation of NF-kB enhances tumor cell survival and proliferation and helps to transform tumor-associated macrophages into tumor-promoting phenotypes (41).

In addition, studies on psoriasis risk in patients with CM are few and inconsistent. The retrospective cohort study of Sam et al. (12) suggested that CM patients were at increased risk for psoriasis. However, the prospective pilot study by Matteo et al.11 suggested that CM patients have a reduced risk of psoriasis. Our results were similar to those of the observational cross-sectional study by Elwood et al. (13), where genetic liability to CM was not associated with psoriasis. More clinical and basic studies are needed to explore the risk and mechanism of psoriasis in CM patients.

### Strengths and limitations

This study had several advantages. To determine the causal relationship between psoriasis and CM without the interference of confounding factors, we performed bidirectional MR analysis. Bidirectional Mendelian randomization analysis is an extension of standard Mendelian randomization analysis. Compared with the latter, the former can investigate whether there is a two-way causal relationship between exposure variables and outcome variables. In addition, genetic variation in CM was derived from the largest available GWAS meta-analysis, ensuring the strength of instruments in MR analyses. We detected and excluded horizontal poly-tropism using MR-PRESSO and MR–Egger regression intercept tests. Finally, there was no overlap between the GWAS summary data for psoriasis and the meta-analysis GWAS summary data for CM, which led to low heterogeneity, low bias, and high accuracy in our study.

We also acknowledge some limitations. First, most of the data used in the analysis mainly came from individuals with European ancestry, which restricts the applicability of our findings to other ethnic groups. Second, due to the lack of detailed clinical data, we were unable to conduct a subgroup analysis, for example, to distinguish between psoriasis vulgaris, arthritic psoriasis, pustular psoriasis, and erythrodermic psoriasis; explore non-linear relationships; or to sort by the severity of psoriasis. A population-based study in Denmark suggested a modestly increased risk of melanoma in patients with mild psoriasis and no increased risk in patients with severe psoriasis and psoriatic arthritis (1). Due to the lack of GWAS data on psoriasis severity classification or strictly defined clinical classification to date, we were unable to use MR methods to study the effect of different severities or different clinical subtypes of psoriasis on CM. Notably, each method used in our analysis had advantages and disadvantages. However, the use of many methods with different assumptions may lead to inconsistent or conflicting outcomes and obscure the conclusions.

## Conclusion

Our results confirmed genetically predicted psoriasis increases risk of CM. Enhanced early screening of cutaneous melanoma in patients with psoriasis may improve clinical burden. In addition, although the reverse MR estimation does not support the causal relationship from CM to psoriasis, it cannot be ruled out that CM has an effect on the incidence of psoriasis; further research is needed to confirm this.

## Data availability statement

The original contributions presented in the study are included in the article/**Supplementary Material**. Further inquiries can be directed to the corresponding author.

## Ethics statement

We did not need further ethical approval for this study because we used data from published studies that had the appropriate ethics committees’ approval.

## Author contributions

NZ: Conceptualization, Methodology, Software, Investigation, Visualization, Writing an original draft. PG: Methodology, Investigation. MT: Investigation, experiment. FY: Methodology, Investigation. TZ: Data Provision, Methodology. RM: Conceptualization, Writing - original draft, Funding acquisition, Supervision. All authors contributed to the article and approved the submitted version.
