# Supplementary material for: Evidence for a causal relationship between psoriasis and cutaneous melanoma: a bidirectional two-sample Mendelian randomized study
Source: Front Immunol. 2023 Jul 12;14:1201167. doi: 10.3389/fimmu.2023.1201167 (PMC10368886; doi:10.3389/fimmu.2023.1201167)
Supplement: Supplementary file 1 [file Table_1.docx]

| **Supplementary Table 1. The heterogeneity of psoriasis instrumental variables.** | | | |
| --- | --- | --- | --- |
| **method** | **Q** | **df** | **P-value** |
| IVW | 26.65314 | 30 | 0.64142 |
| MR-Egger | 26.53369 | 29 | 0.596848 |
| Df, degree of freedom. | | | |

| **Supplementary Table 2. Directional horizontal pleiotropy assessed by intercept term in MR Egger regression of the association between psoriasis and CM.** | | | |
| --- | --- | --- | --- |
| **Variable** | **Egger_intercept** | **SE** | **P-value** |
| PSORIASIS | -0.02253 | 0.065193 | 0.732125 |
| MR, Mendelian randomization; SE, standard error. | | | |

| **Supplement Table 3. MR-PRESSO analysis for the association between psoriasis and CM.** | | | | | | | |
| --- | --- | --- | --- | --- | --- | --- | --- |
| **Outcome** | **MR Analysis** | **Causal Estimate** | **SD** | **T** | **P-value** | **RSS_obs_** | **Global test P-value** |
| CM | MR-PRESSO | 0.226481 | 0.184091 | 1.230263 | 0.228159 | 30.65579 | 0.551 |
| MR, Mendelian randomization; PE, preeclampsia-eclampsia; MR PRESSO: MR Pleiotropy RESidual Sum and Outlier; SD, standard deviation; RSS_obs_, observed residual sum of squares. | | | | | | | |

| **Supplementary Table 4. Instrumental variables used in MR analysis of the association between CM and psoriasis.** | | | | | | | | | | | | | |
| --- | --- | --- | --- | --- | --- | --- | --- | --- | --- | --- | --- | --- | --- |
| **exposure** | **SNP** | **Effect allele** | **Other allele** | **MAF** | **F** | **R^2^ of exposure** | **Exposure (CM)** | | | **Outcome (psoriasis)** | | |  |
|  |  |  |  |  |  |  | **Beta** | **SE** | **P-value** | **Beta** | **SE** | **P-value** |  |
| CM | rs10739221 | C | T | 0.760 | 42.519 | 0.005 | -0.120 | 0.018 | 7.000E-11 | -0.001 | 0.017 | 0.966 |  |
| CM | rs1636744 | T | C | 0.400 | 33.535 | 0.004 | 0.091 | 0.016 | 7.000E-09 | 0.005 | 0.015 | 0.757 |  |
| CM | rs2995264 | G | A | 0.088 | 35.974 | 0.004 | 0.160 | 0.027 | 2.000E-09 | -0.011 | 0.027 | 0.676 |  |
| CM | rs4778138 | G | A | 0.160 | 44.971 | 0.003 | -0.170 | 0.025 | 2.000E-11 | 0.048 | 0.034 | 0.158 |  |
| CM | rs498136 | A | C | 0.320 | 49.484 | 0.006 | 0.120 | 0.017 | 2.000E-12 | 0.026 | 0.017 | 0.119 |  |
| CM | rs6750047 | A | G | 0.430 | 33.535 | 0.004 | 0.092 | 0.016 | 7.000E-09 | -0.033 | 0.015 | 0.030 |  |
| CM | rs6914598 | C | T | 0.320 | 30.149 | 0.004 | 0.100 | 0.018 | 4.000E-08 | -0.043 | 0.016 | 0.008 |  |

| **Supplementary Table 5. The heterogeneity of psoriasis instrumental variables.** | | | |
| --- | --- | --- | --- |
| **method** | **Q** | **df** | **P-value** |
| MR-Egger | 4.840600864 | 5 | 0.435641762 |
| IVW | 4.992306357 | 6 | 0.544800265 |
| Df, degree of freedom. | | | |

| **Supplementary Table 6. Directional horizontal pleiotropy assessed by intercept term in MR Egger regression of the association between CM and psoriasis.** | | | |
| --- | --- | --- | --- |
| **Exposure** | **Egger_intercept** | **SE** | **P-value** |
| CM | -0.025935687 | 0.056268165 | 0.664203684 |
| MR, Mendelian randomization; SE, standard error. | | | |

| **Supplement Table 7. MR-PRESSO analysis for the association between CM and psoriasis.** | | | | | | | |
| --- | --- | --- | --- | --- | --- | --- | --- |
| **outcome** | **MR Analysis** | **Causal Estimate** | **SD** | **T** | **P-value** | **RSS_obs_** | **Global test P-value** |
| psoriasis | MR-PRESSO | -0.048082873 | 0.070180512 | -0.685131412 | 0.51883886 | 6.940252154 | 0.524 |
| MR, Mendelian randomization; PE, preeclampsia-eclampsia; MR PRESSO: MR Pleiotropy RESidual Sum and Outlier; SD, standard deviation; RSS_obs_, observed residual sum of squares. | | | | | | | |
